# Tenascin-C, a Prognostic Determinant of Esophageal Squamous Cell Carcinoma

**DOI:** 10.1371/journal.pone.0145807

**Published:** 2016-01-05

**Authors:** Zhao-Ting Yang, So-Young Yeo, Yong-Xue Yin, Zhen-Hua Lin, Hak-Min Lee, Yan-Hua Xuan, Yan Cui, Seok-Hyung Kim

**Affiliations:** 1 Key Laboratory of Natural Resources of the Changbai Mountain and Functional Molecules, Ministry of Education, Yanbian University, Yanji (133002), China; 2 Department of Pathology, Yanbian University College of Medicine, Yanji (133002), China; 3 Department of Health Science and Technology, Samsung Advanced Institute for Health Science and Technology, Sungkyunkwan University, Seoul, Republic of Korea; 4 Department of Pathology, Samsung Medical Center, Sungkyunkwan University School of Medicine, Seoul (110–745), South Korea; 5 Department of Mathematics, Yanbian University College of Science, Yanji (133002), China; 6 Department of Oncology, Affiliated Hospital of Yanbian University, Yanji, China; INRS, CANADA

## Abstract

**Background:**

Tenascin-C, an adhesion modulatory extracellular matrix molecule, is highly expressed in numerous human malignancies; thus, it may contribute to carcinogenesis and tumor progression. We explored the clinicopathological significance of Tenascin-C as a prognostic determinant of esophageal squamous cell carcinoma (ESCC).

**Methods:**

In ESCC patient tissues and cell lines, the presence of isoforms were examined using western blotting. We then investigated Tenascin-C immunohistochemical expression in 136 ESCC tissue samples. The clinical relevance of Tenascin-C expression and the correlation between Tenascin-C expression and expression of other factors related to cancer-associated fibroblasts (CAFs) were also determined.

**Results:**

Both 250 and 350 kDa sized isoforms of Tenascin-C were expressed only in esophageal cancer tissue not in normal tissue. Furthermore, both isoforms were also identified in all of four CAFs derived from esophageal cancer tissues. Tenascin-C expression was remarkably higher in ESCC than in adjacent non-tumor esophageal epithelium (p < 0.001). Tenascin-C expression in ESCC stromal fibroblasts was associated with patient’s age, tumor (pT) stage, lymph node metastasis, clinical stage, and cancer recurrence. Tenascin-C expression in cancer cells was correlated with an increase in tumor-associated macrophage (TAM) population, cancer recurrence, and hypoxia inducible factor1α (HIF1α) expression. Moreover, Tenascin-C overexpression in cancer cells and stromal fibroblasts was an independent poor prognostic factor for overall survival (OS) and disease-free survival (DFS). In the Cox proportional hazard regression model, Tenascin-C overexpression in cancer cells and stromal fibroblasts was a significant independent hazard factor for OS and DFS in ESCC patients in both univariate and multivariate analyses. Furthermore, Tenascin-C expression in stromal fibroblasts of the ESCC patients was positively correlated with platelet-derived growth factor α (PDGFRα), PDGFRβ, and smooth muscle actin (SMA) expression. The 5-year OS and DFS rates were remarkably lower in patients with positive expressions of both Tenascin-C and PDGFRα (p < 0.001), Tenascin-C and PDGFRβ (p < 0.001), Tenascin-C and SMA (p < 0.001), Tenascin-C and fibroblast activation protein (FAP) (p < 0.001), and Tenascin-C and fibroblast-stimulating protein-1 (FSP1) (p < 0.001) in ESCC stromal fibroblasts than in patients with negative expressions of both Tenascin-C and one of the abovementioned CAF markers.

**Conclusion:**

Our results show that Tenascin-C is a reliable and significant prognostic factor in ESCC. Tenascin-C may thus be a potent ESCC therapeutic target.

## Introduction

ESCC is one of the most common cancers worldwide, and accounts for 90% of esophageal cancer in high-risk populations such as those of North-Central China, northern Iran, and Central Asian countries [1]. Although prevention, diagnosis, and treatment methods for ESCC have greatly progressed, the 5-year survival rate of ESCC patients is only 10%, mainly because the molecular and genetic mechanisms of esophageal cancer remain poorly understood.

Tenascin-C is a complex multifunctional protein that can influence cell behavior directly and indirectly [2]. Since its discovery, Tenascin-C has been reported to be strongly associated with tumorigenesis and cancer progression in many different types of tumors [3, 4]. Functionally, Tenascin-C interacts with fibronectin and can be defined as an anti-adhesive or adhesion-modulating protein; Tenascin-C increases the invasive and metastasis potential of malignant tumors [5].

Although immunohistochemical studies of Tenascin-C expression in ESCC have been performed, the distribution of Tenascin-C in ESCC tissues remains unclear. In this study, expressions of Tenascin-C and CAF markers in 136 human ESCC samples and 20 adjacent non-tumor esophageal mucosa samples were examined using immunohistochemical examinations on tissue microarray slides. The correlations of Tenascin-C expression with clinicopathologic parameters were explored. Furthermore, the role played by Tenascin-C in the prognosis of ESCC was evaluated using Cox regression and Kaplan–Meier analysis. To the best of our knowledge, ours is the first study that showed a correlation between Tenascin-C expression and expression of CAF markers, as well as their clinical significance in ESCC.

## Materials and Methods

### Ethics statement

This research complied with the Helsinki Declaration and was approved by the Human Ethics Committee and the Research Ethics Committee of Samsung Medical Center. All patients provided written informed consent according to institutional guidelines. Patients were informed that the resected specimens were stored by the hospital and potentially used for scientific research, and that their privacy would be maintained. Follow-up survival data were collected retrospectively through medical record analyses (S1 Table).

### Tissue Specimens

A total of 156 formalin-fixed and paraffin-embedded tissue samples including 136 ESCC and 20 adjacent non-tumor esophageal mucosa were obtained from the Department of Pathology at Samsung Medical Center (Seoul, Korea) in accordance with protocols approved by the Institutional Review Board (no. 2014-09-060-001). No patient received preoperative chemotherapy or radiotherapy. Clinical and pathological reports were reviewed for age, sex, tumor size, histological grade, invasion depth (pT), nodal status (pN), and distant metastasis (pM). The median follow-up period was 30 months (range 0–108 months) (S2 Table). The pTNM classification was applied according to guidelines from the 2010 American Joint Committee on Cancer staging manual (AJCC 7th edition).

### Immunohistochemistry

Sections on microslides were deparaffinized with xylene, hydrated using a diluted alcohol series, and immersed in 0.3% H_2_O_2_ in methanol to quench endogenous peroxidase activity. Sections were treated with TE buffer (10 mM Tris and 1 mM EDTA, pH 9.3) at 98°C for 30 min. To reduce non-specific staining, each section was blocked with 4% bovine serum albumin in PBS with 0.1% Tween 20 for 30 min. The sections were then incubated with anti-Tenascin-C monoclonal antibody (1:100, Abcam, UK), anti- SMA (1:100, Millipore, USA), anti-FAP (1:100, Abcam, UK), anti-FSP1 (1:100, Millipore, USA), anti-PDGFRα (1:100, Cell Signaling Technology, USA), anti-PDGFRβ (1:100, Abcam, UK), anti-CD34 (1:100, Abcam, UK), HIF1α (1:100, BD, USA) and anti-CD68 (1:1000, Dako, Denmark) in PBST containing 3 mg/ml goat globulin (Sigma, St. USA) for 60 min at room temperature, followed by three successive washes with buffer. Sections were then incubated with an anti-mouse/rabbit antibody (Envision plus, Dako, Denmark) for 30 min at room temperature. The chromogen used was 3, 3’-diaminobenzidine (Dako, Denmark). Sections were counterstained with Meyer’s hematoxylin. Omitting the primary antibody provided negative controls for immunostaining.

### Semi-quantitative analysis of immunostaining

**Fig 1:**
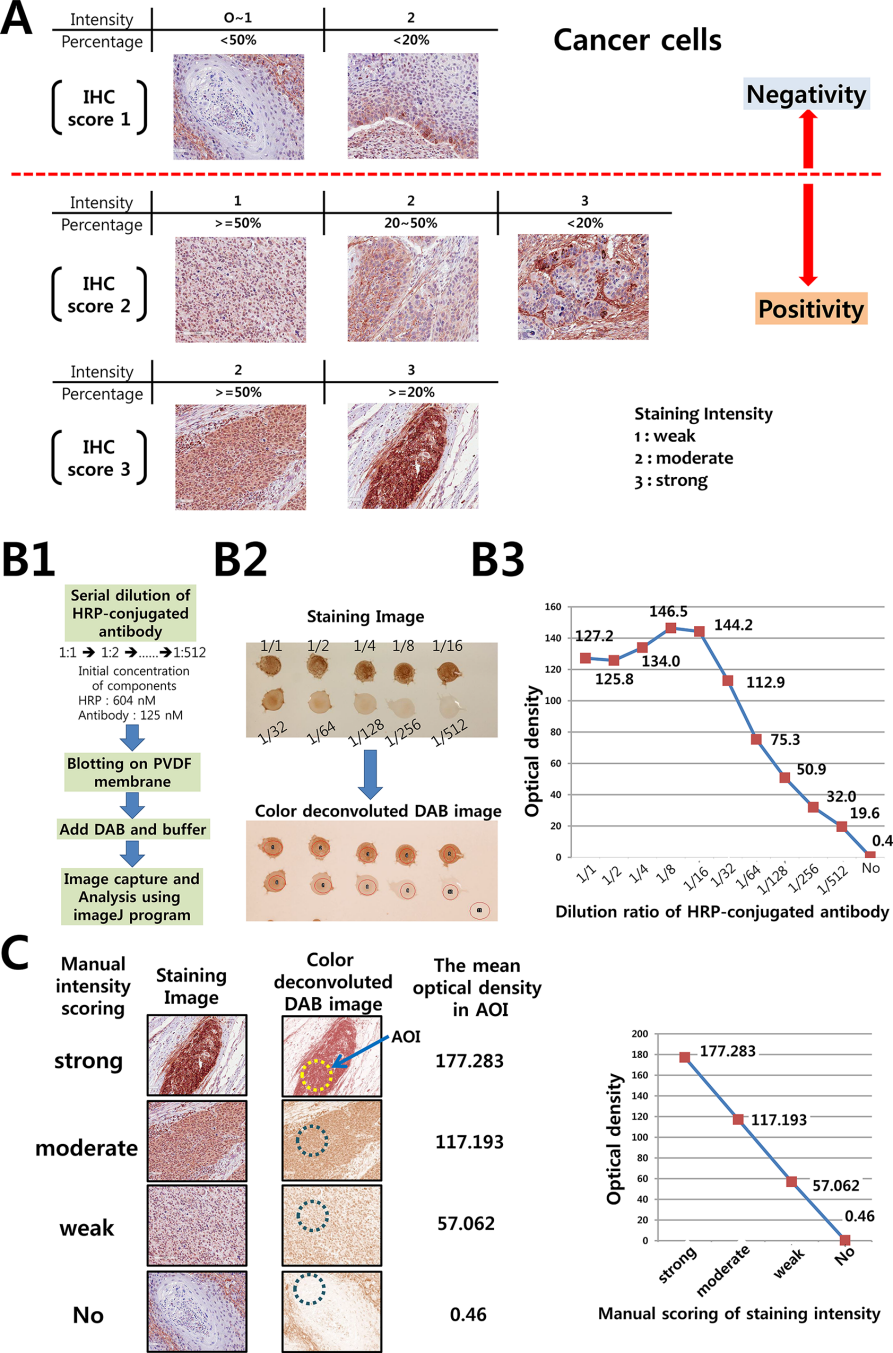
The validation of manual IHC scoring using digital image analysis. (A) The scoring system for manual semi-quantitative evaluation of Tenascin-C immunostaining was illustrated. The representative images for each IHC score of Tenascin-C staining in cancer cells were shown. (B) Relationship between amount of chromogen and optical density measured by digital image analysis was investigated using imageJ program. B1: The controls containing a precisely known concentration of peroxidase were established. B2: The DAB specific images were generated from initial immunostaining images. B3: The optical density of each images was measured by imageJ program and correlation between amount of peroxidase and optical density was shown. (C) Relationship between manual scoring of staining intensity and optical density measured by imageJ program was exhibited.

Two pathologists (YHX & SHK) who did not possess knowledge of the clinical data examined and scored all tissue specimens. As described in detail previously, the staining results were scored semi-quantitatively [6]. In brief, the staining intensity and the proportion of positive cells were measured, and then staining scores were assigned as follows: [IHC score 1], weak staining in <50% or moderate staining in <20% of stromal cells; [IHC score 2], weak staining in ≧50%, moderate staining in 20–50% or strong staining in <20%; [IHC score 3], moderate staining in ≧50% or strong staining in ≧20%. Examples are shown in Fig 1 and S1 Fig. In case of discrepancies, a final score was established by reassessment by both pathologists using a double-headed microscope.

### Measurement of optical density using ImageJ

The optical density of immunostaining was determined using the ImageJ software. As a first step, immunostaining images were acquired in the TIFF format. Then DAB-specific images were generated from initial images by color deconvolution of each image. The color devolution was performed using the set optical density vector for DAB and hematoxylin (H-DAB) in the color deconvolution plugin for ImageJ developed by Ruifrok et al [7]. Finally, the optical density of DAB in the area of interest (AOI) was determined using the ImageJ software.

### Cell lines

TE-1, TE-8, TE-9, TE-10 and TE-11 (ESCC cell lines), were maintained in DMEM with high glucose (Life Technologies, Grand Island, NY) supplemented with 10% heat-inactivated fetal bovine serum (Life Technologies), 100 mg/ml penicillin G, and 50 mg/ml streptomycin (Life Technologies) at 37°C in a humidified atmosphere containing 5% CO2. All cell lines were purchased from the RIEKN BRC Cell Bank (Tsukuba, Japan).

### Isolation and culture of fibroblasts

Human esophageal tumor specimens were obtained from patients undergoing surgery at Samsung Medical Center (Seoul, Korea). An experienced pathologist grossly examined and obtained representative samples of the tumor tissues (CAF and human esophageal CAF). In detail, fresh tissues obtained from two different areas were cut into small pieces and minced using scalpels in a culture dish. Samples were enzymatically dissociated in 20 mL of D/F12+serum medium containing collagenase I in a 37°C incubator for 12−15 h with shaking. After digestion, samples were centrifuged at 700 rpm for 5 min to separate epithelial cells and fibroblasts. Fibroblasts were collected from the supernatant by centrifugation at 800 rpm for 8 min, washed twice with PBS, and cultured in D/F12 medium supplemented with 10% FBS and 1% antibiotics. Samsung Medical Center Biobank provided the biospecimens for this study.

### Western blotting analysis

Cell lysates were produced in RIPA lysis buffer (50 mM Tris pH 7.4, 150 mM NaCl, 1 mM EDTA, 1% Triton x-100, 1% Na-Doc, 0.1% SDS) supplemented with protease inhibitor cocktail (Roche). Cell extracts were quantitated using a BCA protein assay kit (Thermo). Western blot analysis was performed using standard techniques for Tenascin-C and alpha-tubulin. Protein were detected using ECL reagent (Intron, Seoul, Korea).

### Statistical Analysis

Correlations were examined using Pearson’s chi-square test as appropriate. Overall survival (OS) and disease free survival (DFS) were determined using the Kaplan–Meier method and were compared using the log-rank test. Survival was measured from the date of surgery. The Cox proportional hazards model was used for multivariate analysis. Cinicopathologic factors, which were statistically significant in univariated analysis, were included as covariables in multivariate analysis. Hazard ratios (HR) and 95% confidence intervals (CI) were assessed for each factor. All tests were two sided, and P-value of less than 0.05 was considered statistically significant. The statistical analysis was performed using SPSS statistical software (SPSS Inc, Chicago, IL, USA) [6].

## Results

### Semi-quantitative assessment of immunohistochemical staining

Traditionally, immunostaining has been scored semi-quantitatively as–(negative), + (weakly positive), ++ (moderately positive), and +++ (strongly positive). Although this approach may be intuitive, problems arise when staining is heterogeneous, for example, heavy of staining a small number of cells. Therefore, percentages or fractions of positive cells are multiplied by intensity scores and summed [8, 9]. In this study, a similar immunostaining scoring system with minor modification was applied. As shown in Fig 1A, the staining intensity and fraction are first assessed and IHC score is determined using a three-tier scale. After assessing the IHC score, the positivity of each case was decided according to a cut-off point derived from clinical end points, such as recurrence and patient’ survival. In this study, cases with IHC scores of 2 and 3 were regarded as positive for expression of the corresponding protein.

Next, we attempted to evaluate the manually determined grade of staining intensity using digital image analysis. As a first step, a standard curve was created to determine the relationship between optical density and the concentration of chromogen using a peroxidase-conjugated antibody of known concentration. To this end, we created control samples by blotting serially diluted HRP conjugated antibody (DAKO, K5007) initially containing 125 nM of antibody and 604 nM of peroxidase on PVDF membrane (Fig 1B). Immunostaining was completed using DAB as a chromogen. Using the ImageJ software, we obtained DAB-specific images of each blot and determined the optical density of the AOI of each blot (Fig 1B). As in reported by Varghese et al. [10], we confirmed that the relationship between immunogen concentration and optical density was linear at relatively low immunogen concentrations (Fig 1B). Our results are consistent with that previous report [10].

Next, we evaluated our manual scoring of immunostaining intensity by comparing with the optical density measured by the ImageJ software (Fig 1C). We collected the representative images whose intensity was manually graded as weak, moderate, or strong. These images were then deconvoluted to generate DAB-specific images. Optical densities in AOI of each sample was measured and their values were plotted on the Y-axis (Fig 1C). A clear linear relationship between manual scoring and optical density was noted, suggesting that the manual 3-tier intensity scoring system is sufficiently reliable to be used in this study.

### Tenascin-C Expression in ESCCs

**Fig 2:**
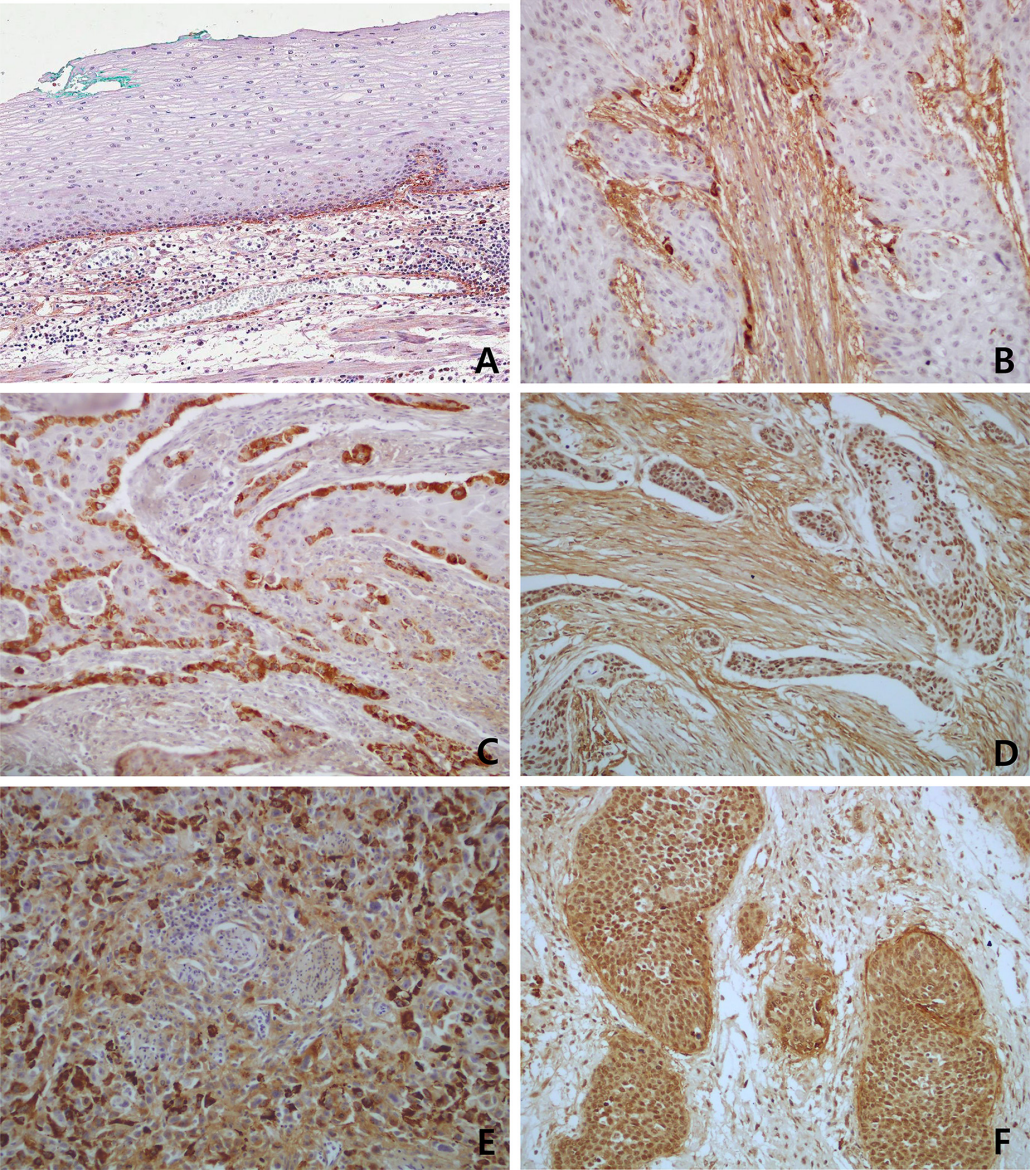
Immunohistochemical staining of Tenascin-C in esophageal squamous cell carcinoma cancer nest and stroma. (A) Tenascin-C was weakly positive in basal layer epithelial cells and stromal fibroblasts of adjacent non-tumor esophageal mucosa. (B) Diffuse and strongly positive Tenascin-C expression was detected in stromal fibroblsts. (C) Strongly positive Tenascin-C expression was detected in cancer invasion front only. (D) Strongly positive Tenascin-C expression was detected in both cancer cells and adjacent stromal fibroblasts. (E) Diffuse and strongly positive Tenascin-C expression was detected in poorly differentiated cancer cells. (F) Diffuse and strongly positive Tenascin-C expression was detected in cancer cells, and a few adjacent macrophage were also positive for Tenascin-C.

Tenascin-C expression (69.1% in stromal fibroblasts and 55.1% in cancer cells) was significantly higher in ESCC tissue samples than in adjacent non-tumor esophageal epithelium (10.0% in stromal fibroblasts and 10.0% in epithelial cells; p < 0.001 and p < 0.001, respectively). Positive signals of Tenascin-C expression were localized mainly in the cytoplasm of cancer cells and stromal fibroblasts. Tenascin-C staining was particularly evident at the cancer cell invasive front (Fig 2). Tenascin-C expression was observed more frequently in stromal fibroblasts (94/136, 69.1%) than in cancer cells (75/136, 55.1%) from patients with ESCC. Of the 136 cases analyzed, 50 (36.8%) showed positive Tenascin-C expression in both cancer cells and stromal fibroblasts, 17 (12.5%) showed negative Tenascin-C expression in both cancer cells and stromal fibroblasts, 25 (18.4%) showed positive Tenascin-C expression in cancer cells only, and 44 (32.4%) showed positive Tenascin-C expression in fibroblasts only.

### Expression profiles of Tenascin-C isoforms in esophageal normal and cancer tissues

**Fig 3:**
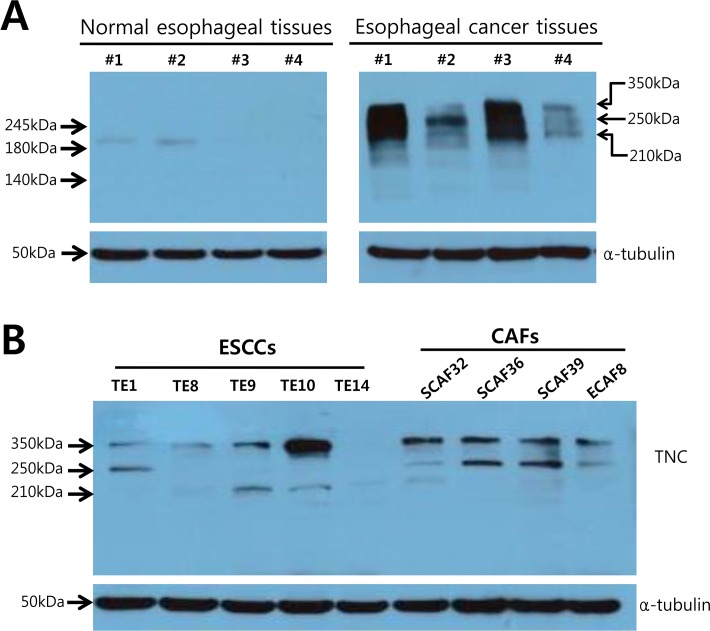
The expression profile of Tenascin-C isoforms in esophageal normal and cancer tissues. (A) Immunoblot analysis of tissue extracts from esophageal cancers and normal tissues using anti-TNC antibody. Four samples of cancer tissues and normal counterparts collected from four cases of esophagectomy were examined. When the extracts were analyzed with this antibody, bands at 350, 250 and 210 kd were intensely labeled in esophageal cancers, but only a single was weakly labeled at 210 kd in normal tissues (left). 350 and 250 kd bands are apparently limited to esophageal cancers (right). (B) Immunoblot analysis of TNC expression in cancer cell lines of ESCC (esophageal squamous cell carcinoma) and ex vivo cultured CAFs (cancer associated fibroblast) derived from esophageal cancer tissue. Five cancer cell lines and four CAFs were collected and TNC expressions were examined.

Tenascin-C exists in multiple isoforms generated by alternative splicing. Therefore we examined the presence of isoforms in ESCC patient tissues and cell lines using western blotting. For this purpose, proteins were extracted from four patient matched normal and esophageal cancer tissues, and then western blot analyses were performed to determine the Tenascin-C isoform profiles and levels. In normal esophageal tissue, only one isoform of Tenascin-C (210 kDa) was identified, whereas at least three isoforms (210 kDa, 250 kDa, and 350 kDa) were found in esophageal cancer tissue (Fig 3A). In addition, the Tenascin-C protein level was markedly higher in esophageal cancer tissues compared with normal tissues (Fig 3A). These results are highly consistent with previous reports of enhanced expressions of multiple isoforms of Tenascin-C in breast and oral cancer tissue, but not in normal tissue [11, 12].

Because cancer tissues contain both cancer and stromal cells, we next determined the protein isoform profiles of Tenascin-C in each cell type. To this end, we established esophageal cancer tissue-derived fibroblast culture and examined protein isoform profiles in five esophageal cancer cell lines and four ex vivo-cultured CAFs by western blotting.

In agreement with the data from cancer tissues shown above, cancer-specific 350 kDa and 250 kDa splicing isoforms of Tenascin-C were detected in four out of five ESCC cell lines. Interestingly, both the 350 kDa and 250 kDa isoforms were identified in all of CAFs derived from esophageal cancer tissues (Fig 3B). These results strongly suggest that cancer-specific large isoforms of Tenascin-C are generated not only by cancer cells but also by CAFs.

### Association of Tenascin-C expression with clinicopathological characteristics

We divided the patients into the following groups to determine the clinical significance of Tenascin-C expression: those who showed Tenascin-C expression in cancer cells and those who showed Tenascin-C expression in stromal fibroblasts. Tenascin-C expression in cancer cells was associated with TAM population (p = 0.043), cancer recurrence (p = 0.014), and HIF1α expression in cancer cells (p = 0.004). Tenascin-C expression in cancer cells was not significantly associated with patient’s age, gender, tumor size, differentiation, pT stage, lymph node metastasis, distant metastasis, clinical stage, microvessel density (MVD), and HIF1α expression in stroma. Tenascin-C expression in stromal fibroblasts was associated with patient’s age (p = 0.018), pT stage (p < 0.001), lymph node metastasis (p = 0.002), clinical stage (p = 0.002), and cancer recurrence (p < 0.001). Tenascin-C expression in stromal fibroblasts was not significantly associated with gender, tumor size, differentiation, distant metastasis, MVD, the number of TAM and HIF1α expression (Table 1). Lymph node metastasis occurred most frequently in Tenascin-C positive in both stroma and cancer cells, followed by Tenascin-C positive in either of stroma and cancer cells and Tenascin-C neative in both stroma and cancer cells (S2A Fig).

**Table 1:** Comparison of clinicopathologic characteristics according to Tenascin-C expression in esophageal squamous cell carcinoma tissues.

Variable		n	Stroma- Tenascin-C(+) n (%)	χ^2^	p value	Cancer cell- Tenascin-C(+)n (%)	χ^2^	p value
**Age (years)**								
	**<65**	34	29(85.3%**)**	5.558	0.018	20(58.8%)	0.248	0.619
	**≥65**	102	65(63.7%**)**			55(53.9%)		
**Gender**								
	**Female**	4	4(100.0%**)**	1.841	0.175	2(50.0%)	0.044	0.834
	**Male**	132	90(68.2%**)**			73(55.3%)		
**Tumor size (cm)**								
	**<4**	88	61(69.3%**)**	0.005	0.945	52(59.1%)	1.568	0.211
	**≥4**	48	33(68.8%**)**			23(47.9%)		
**Differentiation**								
	**Well**	24	14(58.3%**)**	1.761	0.415	12(50.0%)	0.605	0.739
	**Moderately**	86	59(68.6%**)**			49(57.0%)		
	**Poorly**	26	21(80.8%**)**			14(53.8%)		
**pT stage**								
	**1**	27	9(33.3%**)**	27.156	0.000	13(48.1%)	1.849	0.604
	**2**	26	15(57.7%**)**			14(53.8%)		
	**3**	75	64(85.3%**)**			42(56.0%)		
	**4**	8	6(75.0%**)**			6(75.0%)		
**Lymph node metastasis**								
	**Negative**	54	29(53.7%**)**	9.969	0.002	30(55.6%)	0.006	0.938
	**Positive**	82	65(79.3%**)**			45(54.9%)		
**Distant metastasis**								
	**Negative**	118	80(67.8%**)**	0.766	0.382	62(52.5%)	2.337	0.126
	**Positive**	18	14(77.8%**)**			13(72.2%)		
**Clinical stage**								
	**1**	21	7(33.3%**)**	15.266	0.002	12(57.1%)	2.663	0.447
	**2**	48	32(66.7%**)**			23(47.9%)		
	**3**	50	41(82.0%**)**			28(56.0%)		
	**4**	17	14(82.4%**)**			12(70.6%)		
**Microvessel density**								
	**Low**	53	48(90.6%**)**	3.985	0.136	32(60.4%**)**	1.838	0.399
	**Intermediate**	47	22(46.8%**)**			26(55.3%**)**		
	**High**	36	24(66.7%**)**			17(47.2%**)**		
**Tumor associated macrophages**								
	**Low**	56	40(71.4%**)**	0.263	0.608	22(39.3%**)**	5.595	0.018
	**High**	80	54(67.5%**)**			53(66.3%**)**		
**Recurrence**								
	**Yes**	72	65(90.3%)	26.447	0.000	47(65.3%)	6.015	0.014
	**No**	64	29(45.3%)			28(43.8%)		
**HIF1α expression in cancer**								
	**Negative**	70	46(65.7%)	0.616	0.432	30(42.9%)	8.505	0.004
	**Positive**	66	48(72.7%)			45(68.2%)		
**HIF1α expression in stroma**								
	**Negative**	84	54(64.3%)	1.288	0.256	41(48.8%)	3.130	0.077
	**Positive**	52	40(76.0%)			34(65.4%)		

**Fig 4:**
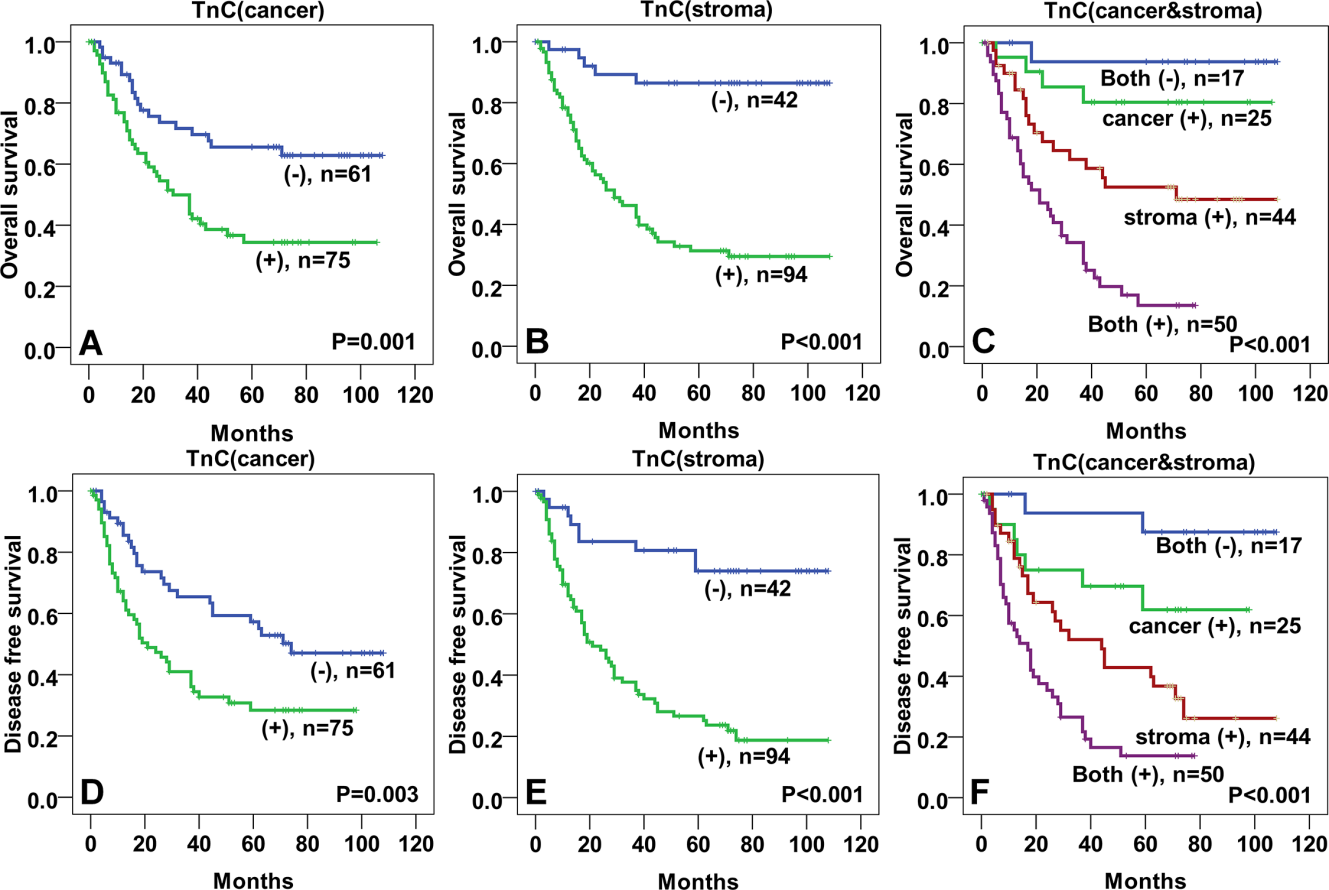
Kaplan- Meier analyses of overall and disease free survival curves for Tenascin-C expression in esophageal squamous cell carcinoma patients. Patients with Tenascin-C positive expressed in cancer cells had lower OS (A) and DFS (D) rates than those with Tenascin-C negative expression. Patients with Tenascin-C positive expression in stromal fibroblast had lower OS (B) and DFS (E) rates than those with Tenascin-C negative expression. Patients with Tenascin-C positive expressed in both cancer cells and stromal fibroblast showed very lowest OS (C) and DFS (F) rate than those with both negative or single positive in ESCC. (TnC: Tenascin-C).

In this study, the survival analysis showed that Tenascin-C expression in stromal fibroblasts and cancer cells was associated with poor OS and DFS. In addition, the 5-year OS and DFS rates of the stroma-Tenascin-C-positive group (37.0% and 29.3%, respectively) were significantly lower than those of the stroma-Tenascin-C-negative group (88.6% and 79.5%, respectively; OS: p < 0.001; DFS: p < 0.001). The 5-year OS and DFS rates in the cancer cell-Tenascin-C-positive group (39.7% and 35.6%, respectively) were also significantly lower than those of the cancer cell-Tenascin-C-negative group (68.3% and 57.1%, respectively; OS: p = 0.001; DFS: p = 0.003). In particular, the 5-year OS and DFS rates of the patients with positive Tenascin-C expression in both cancer cells and stromal fibroblasts (20.0% and 18.0%, respectively) were significantly lower than those of the patients who showed positive Tenascin-C expression in either of the cell types (positive expression in only cancer cells: 83.3% and 70.8%, respectively; positive expression in only stromal fibroblasts: 58.1% and 41.9%, respectively) or those who showed negative expression in both the cell types (94.7% and 89.5%, respectively; OS: p < 0.001; DFS: p < 0.001; Fig 4). Furthermore, the 5-year OS and DFS rates of the Tenascin-C-positive group (positive expression in stroma and cancer, respectively) were significantly lower than those of the Tenascin-C-negative group in lymph node metastasis-negative group. In lymph node metastasis-positive group, the 5-year OS and DFS rates of the Tenascin-C-positive group (positive expression in stroma and cancer, respectively) were also significantly lower than those of the Tenascin-C-negative group (in addition to OS rate of the stroma-Tenascin-C- positive group) (S3 Fig).

In the univariate Cox regression analysis, the following factors were independent poor prognostic factors of both OS and DFS (Table 2): ESCC differentiation (p = 0.022 and p = 0.046, respectively), clinical stage (p < 0.001 and p < 0.001, respectively), Tenascin-C expression status (Tenascin-C in cancer cells: p *=* 0.002 and p *=* 0.004, respectively; Tenascin-C in stromal fibroblasts: p < 0.001 and p < 0.001, respectively), and TAM numbers (p *=* 0.001 and p *=* 0.002, respectively). In the multivariate Cox regression analysis, the following factors were independent poor prognostic factors of both OS and DFS: Tenascin-C expression status (Tenascin-C in cancer cells: p *=* 0.019 and p *=* 0.010, respectively; Tenascin-C in stromal fibroblasts: p *=* 0.002 and p < 0.001, respectively), and TAM numbers (p *=* 0.006 and p *=* 0.005, respectively) (Table 3).

**Table 2:** Univariate Cox proportional hazard model analysis by classification of Tenascin-C expression in esophageal squamous cell carcinoma.

Characteristic	Overall survival			Disease free survival		
	HR	95% CI	p value	HR	95% CI	p value
**Differentiation**			0.022			0.046
**Well**	1.0			1.0		
**Moderate**	2.173	0.988–4.781	0.054	2.220	1.063–4.635	0.034
**Poor**	3.387	1.413–8.117	0.006	2.877	1.239–6.677	0.014
**Clinical stage**			0.000			0.000
**1**	1.0			1.0		
**2**	5.442	1.283–23.085	0.022	3.063	1.069–8.777	0.037
**3**	10.956	2.644–45.405	0.001	5.698	2.037–15.943	0.001
**4**	14.967	3.397–65.949	0.000	9.293	3.119–27.689	0.000
**Age**	1.031	0.603–1.764	0.912	0.930	0.570–1.519	0.773
**Size**	1.464	0.937–2.286	0.094	1.422	0.933–2.169	0.102
**Cancer cell- Tenascin-C**	2.341	1.361–4.026	0.002	2.050	1.262–3.330	0.004
**Stroma-Tenascin-C**	7.778	3.109–19.461	0.000	5.050	2.497–10.214	0.000
**Microvessel density**	0.788	0.413–1.504	0.471	0.818	0.456–1.465	0.499
**Tumor associated macrophages**	2.397	1.410–4.077	0.001	2.171	1.340–3.517	0.002

**Table 3:** Multivariate Cox proportional hazard model analysis by classification of Tenascin-C expression in esophageal squamous cell carcinoma.

Characteristic	Overall survival			Disease free survival		
	HR	95% CI	p value	HR	95% CI	p value
**Differentiation**			0.397			0.669
**Well**	1.0			1.0		
**Moderate**	1.508	0.426–5.341	0.524	1.505	0.488–4.639	0.477
**Poor**	2.334	0.601–9.065	0.221	1.786	0.502–6.354	0.370
**Clinical stage**			0.078			0.107
**1**	1.0			1.0		
**2**	2.875	0.334–24.786	0.337	1.781	0.472–6.715	0.394
**3**	4.889	0.574–41.623	0.146	2.037	0.492–8.427	0.326
**4**	8.789	1.009–76.571	0.049	4.138	1.058–16.181	0.041
**Age**	1.191	0.517–2.744	0.682	1.186	0.572–2.461	0.647
**Size**	1.287	0.619–2.674	0.499	1.216	0.632–2.339	0.559
**Cancer cell-Tenascin-C**	2.423	1.157–5.072	0.019	2.485	1.244–4.964	0.010
**Stroma-Tenascin-C**	6.010	1.931–18.706	0.002	5.398	2.093–13.920	0.000
**Microvessel density**	1.182	0.507–2.755	0.699	1.049	0.474–2.325	0.905
**Tumor associated macrophages**	2.665	1.332–5.331	0.006	2.501	1.327–4.713	0.005

### Correlation of Tenascin-C expression with CAF markers expression

Five CAF markers, including platelet-derived growth factor α (PDGFRα), PDGFRβ, smooth muscle actin (SMA), fibroblast activation protein (FAP), and fibroblast-stimulating protein-1 (FSP1), were heterogeneously expressed in stromal fibroblasts of 136 ESCC tissue samples. Tenascin-C expression in ESCC stromal fibroblasts was associated with the expression of PDGFRα (p *=* 0.025), PDGFRβ (p *=* 0.013), and SMA (p *=* 0.049). Tenascin-C expression was not significantly associated with FAP and FSP1 expression (Table 4). Furthermore, lymph node metastasis occurred more frequently when ESCC stromal fibroblasts showed positive expressions of both Tenascin-C and PDGFRβ (p = 0.038), Tenascin-C and SMA (p = 0.038), Tenascin-C and FAP (p < 0.001), Tenascin-C and FSP1 (p = 0.014), than when stromal fibroblasts were negative of both Tenascin-C and CAF markers (S2B–S2F Fig).

**Table 4:** The association between expression of Tenascin-C and that of cancer associated fibroblast markers in esophageal squamous cell carcinoma stromal tissues.

Variable	n	Tenascin C(+)n (%)	χ^2^	p value	R
**PDGFRα**					
**Negative**	16	7 (43.8%)	5.010	0.025	0.195
**Positive**	120	87 (72.5%)			
**PDGFRβ**					
**Negative**	63	37 (58.7%)	6.125	0.013	0.215
**Positive**	73	57 (78.1%)			
**SMA**					
**Negative**	31	17 (54.8%)	3.878	0.049	0.172
**Positive**	105	77 (73.3%)			
**FAP**					
**Negative**	50	33 (66.0%)	0.460	0.498	0.059
**Positive**	86	61 (70.9%)			
**FSP1**					
**Negative**	35	26 (74.3%)	0.632	0.427	-0.070
**Positive**	101	68 (67.3%)			

**Fig 5:**
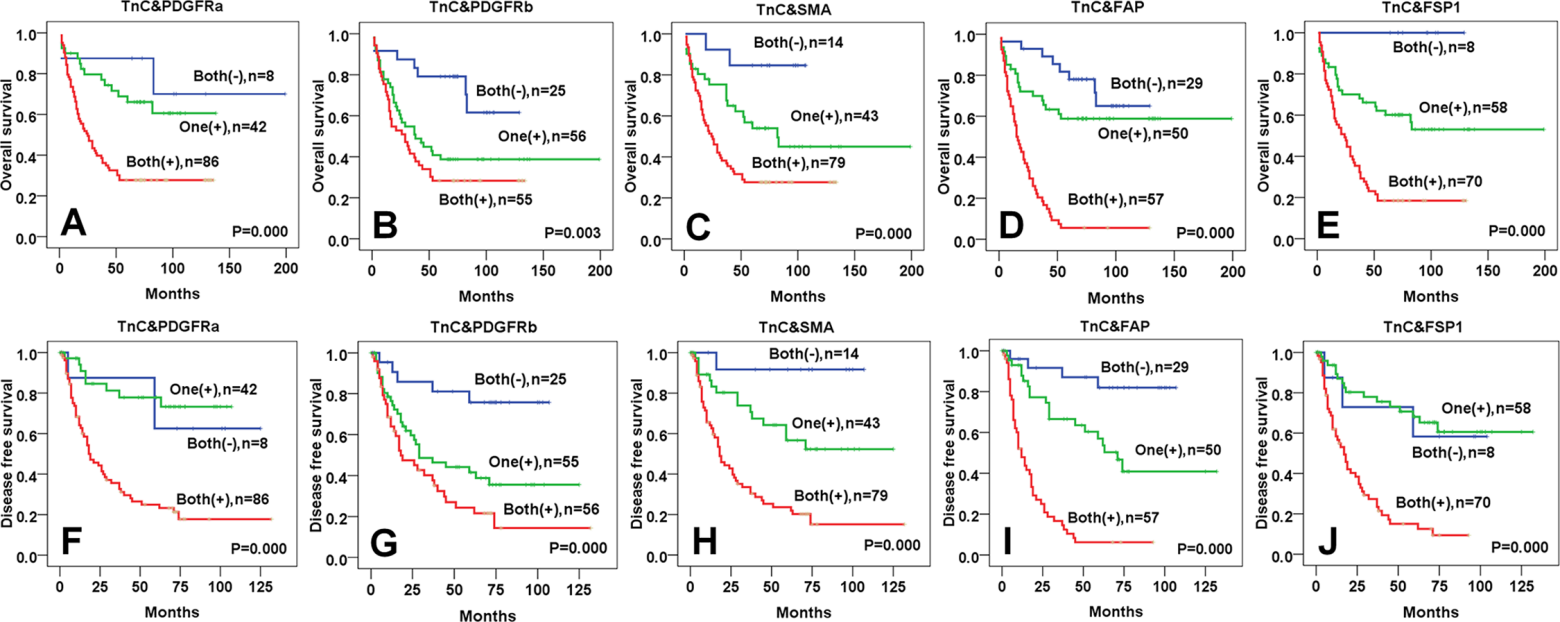
Overall and disease free survival curves using the Kaplan- Meier analyses for Tenascin-C with cancer associated fibroblast markers expression in esophageal squamous cell carcinoma stromal tissues. OS and DFS rates were lower in ESCC stromal fibroblasts which were positive expression of both Tenascin-C and PDGFRα (A, F), Tenascin-C and PDGFRβ (B, G), Tenascin-C and SMA (C, H), Tenascin-C and FAP (D, I), Tenascin-C and FSP1 (E, J), and higher in stromal fibroblasts which were negative expression of both Tenascin-C and CAF markers. (TnC: Tenascin-C).

The 5-year OS and DFS rates of patients with positive expressions of both Tenascin-C and PDGFRα (OS rate, p < 0.001 and DFS rate, p < 0.001, respectively), Tenascin-C and PDGFRβ (OS rate, p *=* 0.003 and DFS rate, p < 0.001, respectively), Tenascin-C and SMA (OS rate, p < 0.001 and DFS rate, p *=* 0.000, respectively), Tenascin-C and FAP (OS rate, p < 0.001 and DFS rate, p < 0.001, respectively), and Tenascin-C and FSP1 (OS rate, p < 0.001 and DFS rate, p < 0.001, respectively) in ESCC stromal fibroblasts were significantly lower than patients with negative expressions of both Tenascin-C and CAF markers (Fig 5, Table 5).

**Table 5:** The overall survival and disease free survival rates of patients with expression of Tenascin-C (TNC) and cancer associated fibroblast markers in esophageal squamous cell carcinoma stromal tissues. Both (-): negative for both markers, One (+): positive for either of both markers, Both (+): positive for both markers.

Variable		n	Alive (overall survival rate)	p value	Alive (disease free survival rate)	p value
**TNC &**	**Both (-)**	8	6 (75.0%)	0.000	5 (62.5%)	0.000
**PDGFRα**	**One (+)**	42	27 (64.3%)		33 (78.6%)	
	**Both (+)**	86	24 (27.9%)		24 (27.9%)	
**TNC &**	**Both (-)**	25	18 (72.0%)	0.003	20(80.0%)	0.000
**PDGFRβ**	**One (+)**	56	23 (41.1%)		25 (44.6%)	
	**Both (+)**	55	16 (29.1%)		17 (30.9%)	
**TNC &**	**Both (-)**	14	12 (85.7%)	0.000	13 (92.9%)	0.000
**SMA**	**One (+)**	43	22 (51.2%)		27 (62.8%)	
	**Both (+)**	79	22(27.8%)		20 (25.3%)	
**TNC &**	**Both (-)**	29	21 (72.4%)	0.000	25 (86.2%)	0.000
**FAP**	**One (+)**	50	30 (60.0%)		28 (56.0%)	
	**Both (+)**	57	3 (5.3%)		7 (12.3%)	
**TNC &**	**Both (-)**	8	8 (100.0%)	0.000	5 (62.5%)	0.000
**FSP1**	**One (+)**	58	33 (56.9%)		41 (70.7%)	
	**Both (+)**	70	13 (18.6%)		14 (20.0%)	

## Discussion

In this study, we found that overexpression of Tenascin-C in stromal fibroblasts and cancer cells was an independent predictor of poor prognosis in ESCC patients. In addition, Tenascin-C expression was positively correlated with the expression of CAF markers such as PDGFRα, PDGFRβ, and SMA in ESCC.

Tenascin-C has been suggested to be a predictor or biomarker of tumor invasion, metastasis, and survival in numerous malignant cancers, and has been investigated as a therapy target [3, 13–15]. A higher level of Tenascin-C expression was found in patients with metastatic hepatocellular carcinoma (HCC), and overexpression of Tenascin-C was highly correlated with poor prognosis in HCC patients [13]. In addition, Tenascin-C expression is associated with lymph node metastasis in breast, colon, liver, and oral squamous cell carcinoma [3]. Some studies showed that the large isoform of Tenascin-C was overexpressed in non-small cell lung cancer that showed recurrence, suggesting that Tenascin-C is crucial for the progression and spread of cancer [14]. In a mouse model, Tenascin-C knockdown dramatically inhibited lung metastasis and colonization by breast cancer cells [15]. In the present study, we also found that Tenascin-C expression in ESCC stromal fibroblasts was related to pT stage, lymph node metastasis, clinical stage, and cancer recurrence. These findings suggest that Tenascin-C plays a role in ESCC recurrence, invasion, and metastasis. Therefore, we performed a survival analysis to determine whether Tenascin-C can be used as an important independent biomarker in ESCC. The results of the survival analysis showed that overexpression of Tenascin-C in ESCC cancer cells as well as in stromal fibroblasts was an independent poor prognostic factor of both OS and DFS. Especially, patients with Tenascin-C expression in both cancer cells and stromal fibroblasts showed apparently reduced OS and DFS rates. Despite the difference in the organs involved and cancer types, our result is highly consistent with the results of previous studies in which patients with breast cancer and HCC with high expression levels of Tenascin-C showed a poor survival prognosis [13, 16].

In the present study, we found that HIF1α expression in cancer cells was positively associated with the expression of Tenascin-C in cancer cells, and this finding suggests that hypoxia promotes Tenascin-C expression in ESCC cells. Hypoxia is a characteristic of abnormal tumor microenvironment, and is intrinsically linked to the formation of neovasculature and clinically associated with metastasis and poor patient outcome; furthermore, it can induce Tenascin-C expression [17]. On the other hand, infiltration of TAMs is correlated with poor prognosis in ESCC [6]. The Tenascin-C-positive areas in macrometastases of breast cancer to the lungs were infiltrated with myofibroblasts and macrophages [14]. In agreement with this result, in our study, Tenascin-C expression in cancer cells was associated with increased TAM population in ESCC, suggesting that hypoxia and TAM infiltration along with high Tenascin-C expression in areas around the cancer cells play a very important role in metastasis and progression of ESCC. However, further study is required to determine the more specific relationship between Tenascin-C expression and hypoxia or TAM infiltration in ESCC.

CAFs are most often denoted by the expression of PDGFRα, PDGFRβ, SMA, FAP, and FSP1 [18–20]. Nevertheless, none of the markers in specific individually labels all CAFs or clearly distinguishes CAFs from normal fibroblasts or other closely related cell types. Therefore, for a general classification of these various cell types, a combination of markers must be used. Ha SU et al. showed that some of the individual CAF markers were significant prognostic predictors of ESCC. For example, PDGFRα expression in CAF was an essential factor in the prognosis of ESCC; PDGFRβ expression was associated with poorly differentiated tumors; SMA expression in ESCC stromal fibroblasts was associated with a large size of the tumor, advanced pT stage, lymph node metastasis, and poor prognosis. FAP expression was associated with a high mortality rate. Furthermore, ESCC patients with positive FSP1 expression were older and showed shorter survival rates [6]. In colorectal cancer, the frequency of Tenascin-C expression in immature stroma is high, and Tenascin-C expression represents the components of extracellular matrix produced mainly by myofibroblasts at the edge of tumor invasion [21]. However, the relationship between Tenascin-C expression and esophageal CAF marker expression has not yet been explored. The present study showed that Tenascin-C expression in stromal fibroblasts was correlated with the expression of PDGFRα, PDGFRβ, and SMA. In particular, statistically significant lower 5-year OS and DFS rates were observed in patients with positive expressions of Tenascin-C and CAF markers in ESCC stromal fibroblasts than in patients with negative expressions of Tenascin-C and CAF markers. Our results suggest that the immunohistochemical expression of Tenascin-C along with other CAF markers could be a useful marker for the selection of patients with a high risk of unfavorable clinical outcomes and for stratification of these patients for improved therapeutic strategies.

In conclusion, a high expression of Tenascin-C could be a useful CAF marker for the prediction of short-term survival of ESCC patients. Moreover, Tenascin-C could be a novel therapeutic marker for selective targeting of stromal fibroblasts and cancer cells in ESCC.

## Supporting Information

S1 Fig Comparison of lymph node metastasis frequency according to Tenascin-C and cancer associated fibroblast markers expression in ESCC tissues. Lymph node metastasis occurred most frequently in both stroma and cancer cells group as Tenascin-C positive expression (A), and in ESCC stromal fibroblasts as positive expression of both Tenascin-C and PDGFRα (B), Tenascin-C and PDGFRβ (C), Tenascin-C and SMA (D), Tenascin-C and FAP (E), Tenascin-C and FSP1 (F).
(TIF)

S2 Fig The frequency of Nodal metastasis in various subgroup of esophageal cancer patients divided by expression of Tenascin-C and other markers. Lymph node metastasis occurred most frequently in Tenascin-C negative in both stroma and cancer cells, followed by Tenascin-C positive in stroma or cancer cells and Tenascin-C negative in both stroma and cancer cells (S2A Fig). And nodal metastasis occurred more frequently when ESCC stromal fibroblasts showed expressions of both Tenascin-C and PDGFRβ (p = 0.038), Tenascin-C and SMA (p = 0.038), Tenascin-C and FAP (p < 0.001), Tenascin-C and FSP1 (p = 0.014), than when stromal fibroblasts were negative for both Tenascin-C and CAF markers (S2-F Fig).
(TIFF)

S3 Fig Kaplan- Meier analyses of overall and disease free survival curves for Tenascin-C expression in lymph node metastasis negative and positive group. The 5-year OS (A, B) and DFS (E, F) rates of the Tenascin-C-positive group (positive expression in cancer cells and stroma, respectively) were significantly lower than those of the Tenascin-C-negative group in lymph node metastasis-negative group. In lymph node metastasis-positive group, the 5-year OS (C, D) and DFS (G, H) rates of the Tenascin-C-positive group (positive expression in cancer cells and stroma, respectively) were also significantly lower than those of the Tenascin-C-negative group (in addition to OS rate of the stroma-Tenascin-C- positive group).
(TIFF)

S1 Table Clinical characteristics of 136 patients with esophageal squamous cell carcinoma. (DOCX)

S2 Table Clinical characteristics of 20 patients with adjacent non-tumor esophageal mucosa. (DOCX)
