# Supplementary figures and images for: Tenascin-C, a Prognostic Determinant of Esophageal Squamous Cell Carcinoma
Source: PLoS One. 2016 Jan 5;11(1):e0145807. doi: 10.1371/journal.pone.0145807 (PMC4701415; doi:10.1371/journal.pone.0145807)

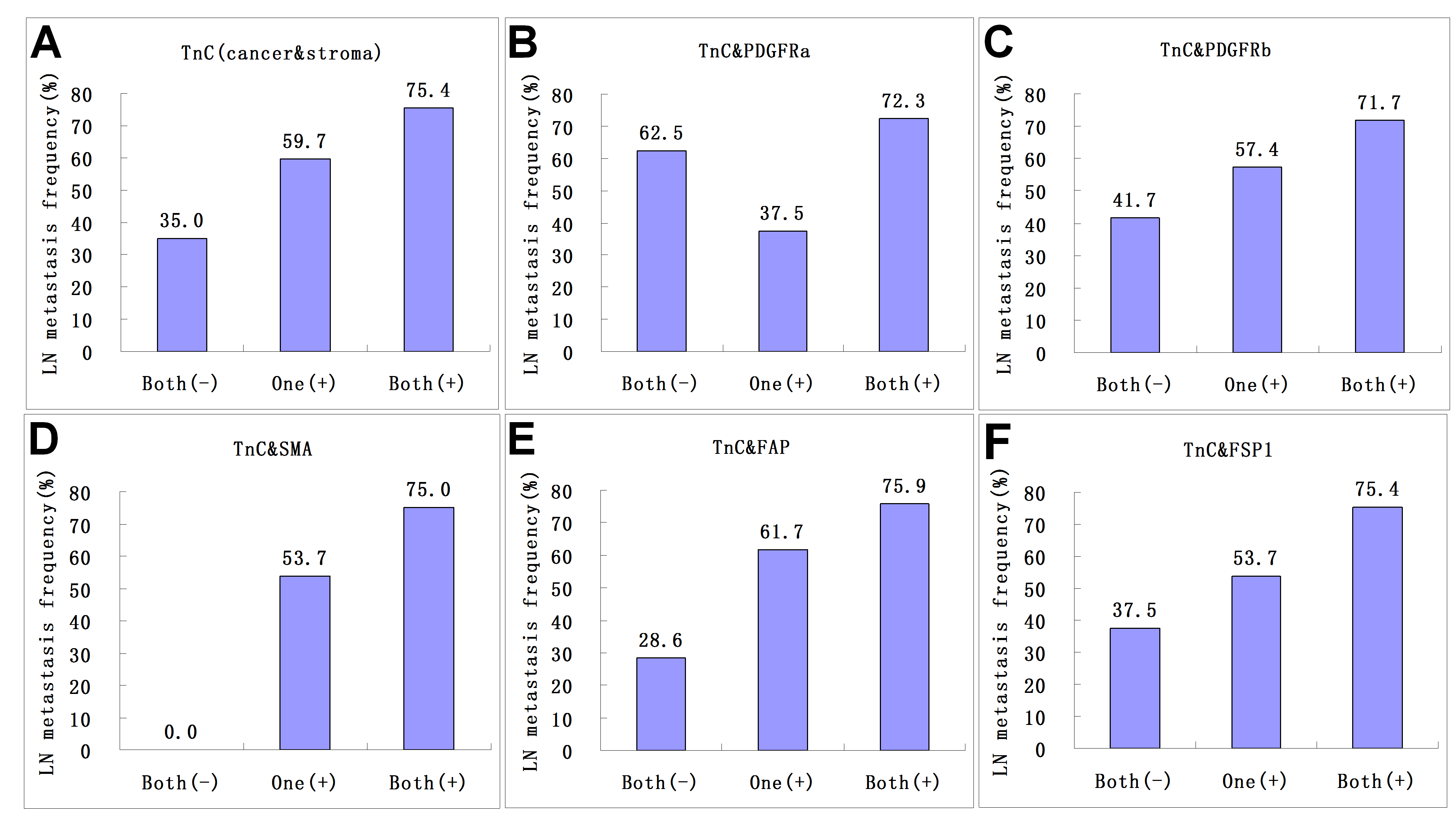

Supplement: S2 Fig — Lymph node metastasis occurred most frequently in Tenascin-C negative in both stroma and cancer cells, followed by Tenascin-C positive in stroma or cancer cells and Tenascin-C negative in both stroma and cancer cells (S2A Fig). And nodal metastasis occurred more frequently when ESCC stromal fibroblasts showed expressions of both Tenascin-C and PDGFRβ (p = 0.038), Tenascin-C and SMA (p = 0.038), Tenascin-C and FAP (p < 0.001), Tenascin-C and FSP1 (p = 0.014), than when stromal fibroblasts were negative for both Tenascin-C and CAF markers (S2-F Fig). (TIFF) [file pone.0145807.s002.tiff]

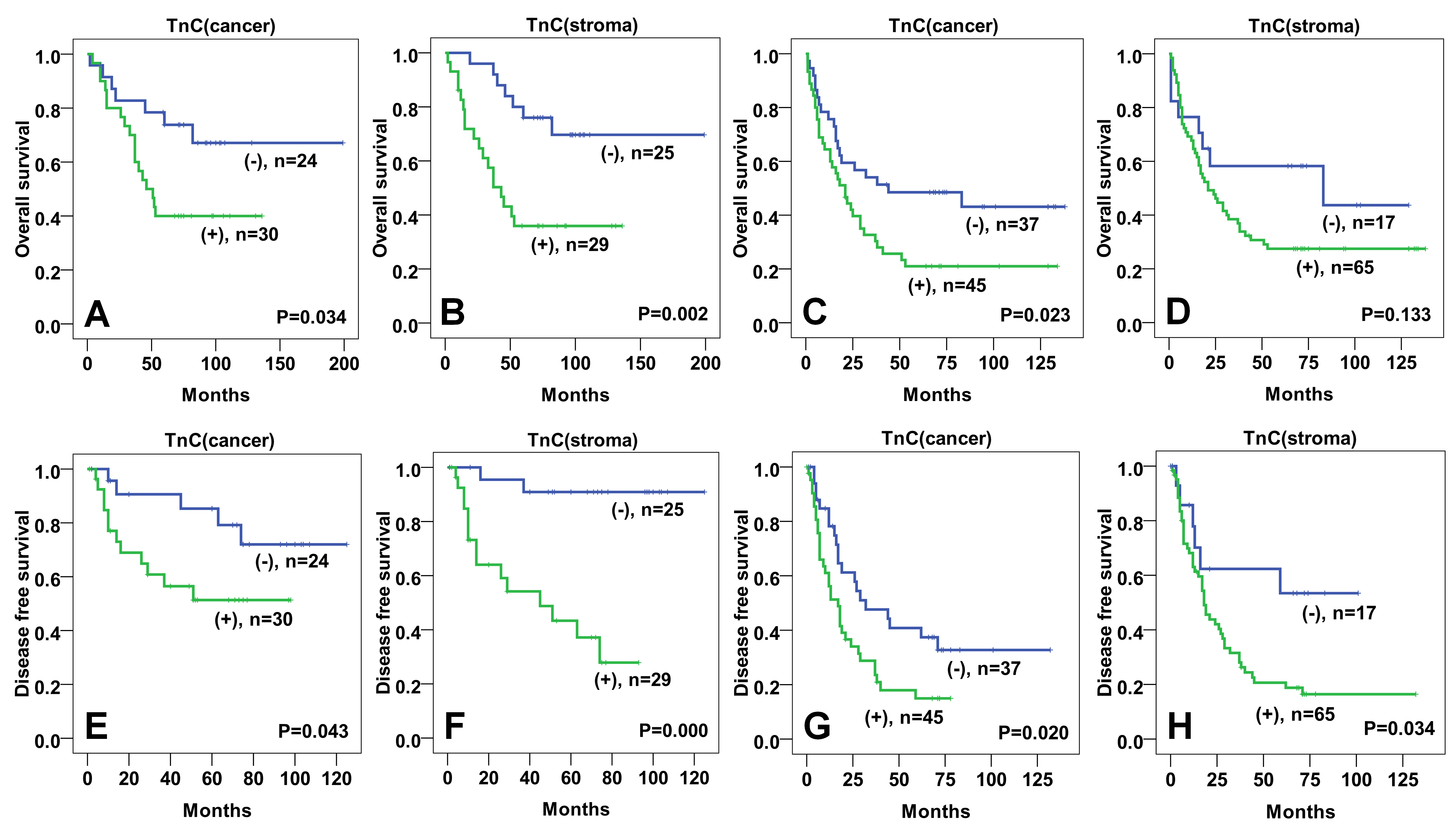

Supplement: S3 Fig — The 5-year OS (A, B) and DFS (E, F) rates of the Tenascin-C-positive group (positive expression in cancer cells and stroma, respectively) were significantly lower than those of the Tenascin-C-negative group in lymph node metastasis-negative group. In lymph node metastasis-positive group, the 5-year OS (C, D) and DFS (G, H) rates of the Tenascin-C-positive group (positive expression in cancer cells and stroma, respectively) were also significantly lower than those of the Tenascin-C-negative group (in addition to OS rate of the stroma-Tenascin-C- positive group). (TIFF) [file pone.0145807.s003.tiff]
