# Supplementary material for: Tenascin-C, a Prognostic Determinant of Esophageal Squamous Cell Carcinoma
Source: PLoS One. 2016 Jan 5;11(1):e0145807. doi: 10.1371/journal.pone.0145807 (PMC4701415; doi:10.1371/journal.pone.0145807)
Supplement: S1 Table — (DOCX) [file pone.0145807.s004.docx]

**S1 Table. Clinical characteristics of 136 patients with esophageal squamous cell carcinoma**

| **Variable** | **n** |
| --- | --- |
| **Age (years)** |  |
| **<65** | 34 |
| **≥65** | 102 |
| **Gender** |  |
| **Female** | 4 |
| **Male** | 132 |
| **Tumor size (cm)** |  |
| **<4** | 88 |
| **≥4** | 48 |
| **Differentiation** |  |
| **Well** | 24 |
| **Moderately** | 86 |
| **Poorly** | 26 |
| **pT stage** |  |
| **1** | 27 |
| **2** | 26 |
| **3** | 75 |
| **4** | 8 |
| **Lymph node metastasis** |  |
| **Negative** | 54 |
| **Positive** | 82 |
| **Distant metastasis** |  |
| **Negative** | 118 |
| **Positive** | 18 |
| **Clinical stage** |  |
| **1** | 21 |
| **2** | 48 |
| **3** | 50 |
| **4** | 17 |
| **Microvessel density** |  |
| **Low** | 53 |
| **Intermediate** | 47 |
| **High** | 36 |
| **Tumor associated macrophages** |  |
| **Low** | 56 |
| **High** | 80 |
| **Recurrence** |  |
| **Yes** | 72 |
| **No** | 64 |
| **HIF1α expression in cancer** |  |
| **Negative** | 70 |
| **Positive** | 66 |
| **HIF1α expression in stroma** |  |
| **Negative** | 84 |
| **Positive** | 52 |
| **Survival status** |  |
| **Alive** | 56 |
| **Dead** | 80 |
