# Supplementary material for: Tenascin-C, a Prognostic Determinant of Esophageal Squamous Cell Carcinoma
Source: PLoS One. 2016 Jan 5;11(1):e0145807. doi: 10.1371/journal.pone.0145807 (PMC4701415; doi:10.1371/journal.pone.0145807)
Supplement: S2 Table — (DOCX) [file pone.0145807.s005.docx]

**S2 Table. Clinical characteristics of 20 patients with adjacent non-tumor esophageal mucosa**

| **Variable** | **n** |
| --- | --- |
| **Age (years)** |  |
| **<65** | 5 |
| **≥65** | 15 |
| **Gender** |  |
| **Female** | 4 |
| **Male** | 16 |
